# Intravitreal ranibizumab for choroidal neovascularization in a patient with angioid streaks and multiple evanescent white dots

**DOI:** 10.1186/s12886-016-0307-0

**Published:** 2016-07-26

**Authors:** Alfredo Pece, Davide Allegrini, Stelios Kontadakis, Giuseppe Querques, Luca Rossetti

**Affiliations:** 1 Department of Ophthalmology, Melegnano Hospital, Via Pandina 1, 20077 Vizzolo Predabissi, Milan, Italy; 2 University Paris XII, Creteil, France; 3 Eye Clinic, San Paolo Hospital, University of Milan, Milan, Italy

**Keywords:** Angioid streaks, Choroidal neovascularization, Multiple evanescent white dots, Ranibizumab

## Abstract

**Background:**

To report a patient with angioid streaks (ASs) and coincident multiple evanescent white dot syndrome (MEWDS) who developed choroidal neovascularization (CNV).

**Case presentation:**

A 20-year-old woman presented with reduced vision (20/100) in her left eye (LE). Based on a complete ophthalmologic examination the patient was diagnosed with ASs and coincident MEWDS. Two weeks later best-corrected visual acuity (BCVA) improved up to 20/25 and the MEWDS findings almost disappeared. Two months later BCVA dropped again (20/100) due to the development of CNV which was treated by a single intravitreal injection of ranibizumab (0.5 mg/0.05 mL). One month after this BCVA improved up to 20/40, and there was regression of the CNV. There was no need for retreatment at the last follow-up visit, 1 year after the ranibizumab injection, when the patient showed further recovery of BCVA up to 20/25.

**Conclusions:**

In this case of ASs, MEWDS completely resolved after 2 weeks, but 2 months later CNV developed. A single intravitreal injection of ranibizumab had a long-lasting effect. Larger series are necessary to clarify the pathogenesis of CNV in such cases and the role of intravitreal ranibizumab.

## Background

Angioid streaks (ASs) are irregular linear breaks in the Bruch’s membrane that typically taper from the optic disk, usually associated with various systemic diseases such as Ehlers-Danlos syndrome, Paget disease, and pseudoxanthoma elasticum (PXE) [1]. The main vision-threatening complication of AS is choroidal neovascularization (CNV) which develops in 72–86 % of eyes [2, 3].

Multiple evanescent white dot syndrome (MEWDS) is a benign inflammatory disorder of unknown etiology that usually affects young women [4]. >It involves unilateral small, transient white dots at the level of the outer retina, the retinal pigment epithelium, and the inner choroid, which have a characteristic appearance on fluorescein angiography (FA) and indocyanine green angiography (ICGA). Patients present various symptoms such as photopsias, blurred vision, sudden decrease in visual acuity and visual field defects, either temporally or paracentrally [5]. The prognosis is generally very good, even though the rare occurrence of CNV may lead to permanent visual loss [6–10].

Here we report a patient with ASs, diagnosed with coincident MEWDS. Two months after the diagnosis of MEWDS the patient developed CNV, which was effectively treated by intravitreal ranibizumab (Lucentis®, Novartis, Basel, Switzerland).

## Case presentation

**Fig. 1:**
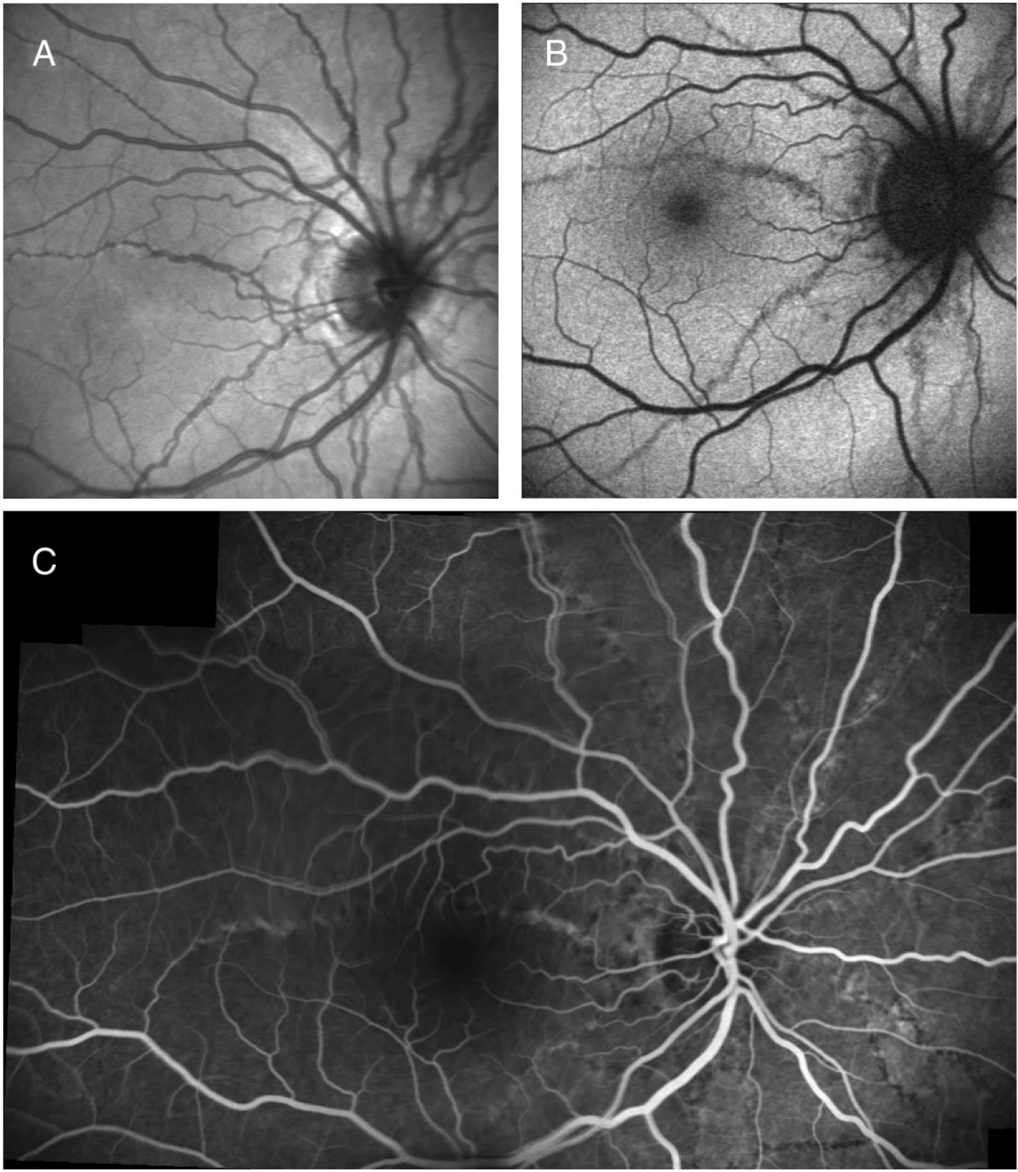
Infrared (IF) (**a**), Autofluorescence (AF) (**b**) and fluorescein angiography (FA) (**c**) images of the right eye (RE) show the angioid streaks (ASs) with no evidence of inflammation, and choroidal neovascularization (CNV)

**Fig. 2:**
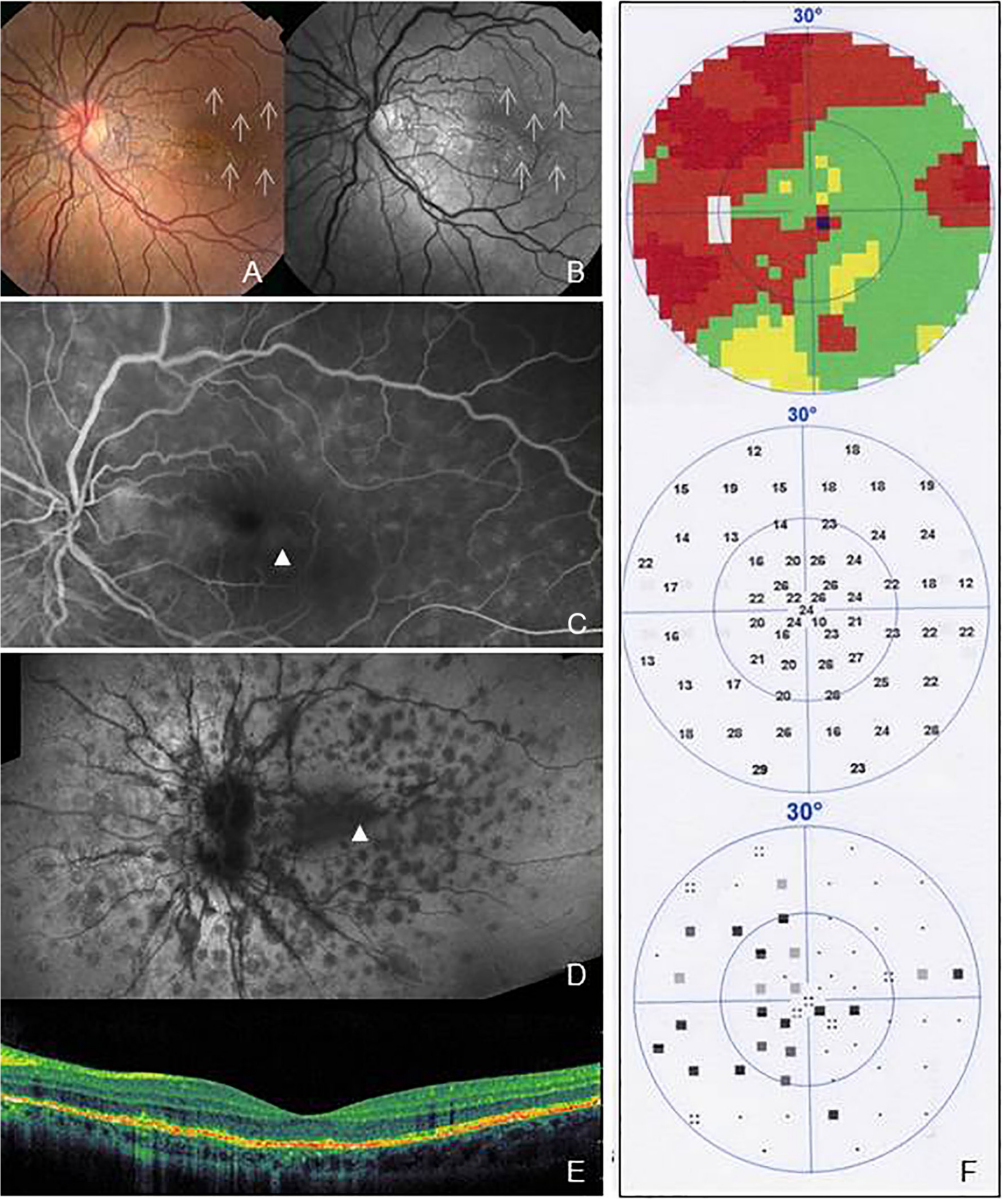
Fundus color (**a**) and red-free (**b**) photographs of the left eye (LE) show multiple discrete grey-white lesions (*dots*) scattered throughout the fundus, with a granular appearance in the macula (**a** and **b**). Fluorescein angiography (FA) late frame indicates mild optic disk leakage with some hyperfluorescent changes scattered over the fundus (**c**). Indocyanine green angiography (ICGA) discloses late hypofluorescent lesions scattered at the posterior pole and in the mid-periphery (**d**). Spectral-domain optical coherence tomography (SD-OCT) macular scans show disruption in the photoreceptor layer (**e**). Automated static threshold perimetry reveals visual field defects mainly located paracentrally and temporally (**f**)

A 20-year-old woman with diagnosis of ASs was referred to our department for reduced vision in her left eye (LE) of 2 weeks onset; she was negative for pseudoxanthoma elasticum or any other systemic disease. The patient signed a comprehensive consent form according to Good Clinical Practice guidelines, before proceeding with all examinations and treatments. Her best-corrected visual acuity (BCVA) was 20/20 in the right eye (RE) and 20/100 in the LE, with no signs of inflammation in the anterior chamber and vitreous of either eye. Fundus biomicroscopy revealed ASs in both eyes (BE). RE had no evidence of inflammation at the fundus observation (Fig. 1). Interestingly, only the LE had multiple discrete grey-white lesions (dots) scattered over the fundus, from the paramacular area up to the mid-periphery, and the macula had a granular appearance (Fig. 2a, b). Fluorescein angiography (FA) (HRA, Heidelberg Engineering, Heidelberg, Germany) indicated mild optic disk leakage with some hyperfluorescent changes scattered throughout the fundus (paramacular area and mid-periphery). No CNV was detected (Fig. 2c). Indocyanine green angiography (ICGA) (HRA, Heidelberg Engineering, Heidelberg, Germany) disclosed late hypofluorescent lesions scattered at the posterior pole and in the mid-periphery (Fig. 2d); we interpreted these as signs of zonal outer retinal inflammation. Spectral-domain optical coherence tomography (SD-OCT) macular scans showed disruption in the photoreceptor layer (Fig. 2e). Automated static threshold perimetry indicated visual field defects mainly located paracentrally and temporally (Fig. 2f).

On the basis of all these findings a diagnosis of ASs and coincident MEWDS was made. The patient was prescribed oral prednisone (1 mg/kg) for 7 days then half the dosage for another 7 days.

Two weeks later, LE BCVA improved up to 20/25, with resolution of the MEWDS findings, except for the granular appearance at the macula; RE BCVA was 20/20 with no signs of inflammation at the fundus evaluation.

**Fig. 3:**
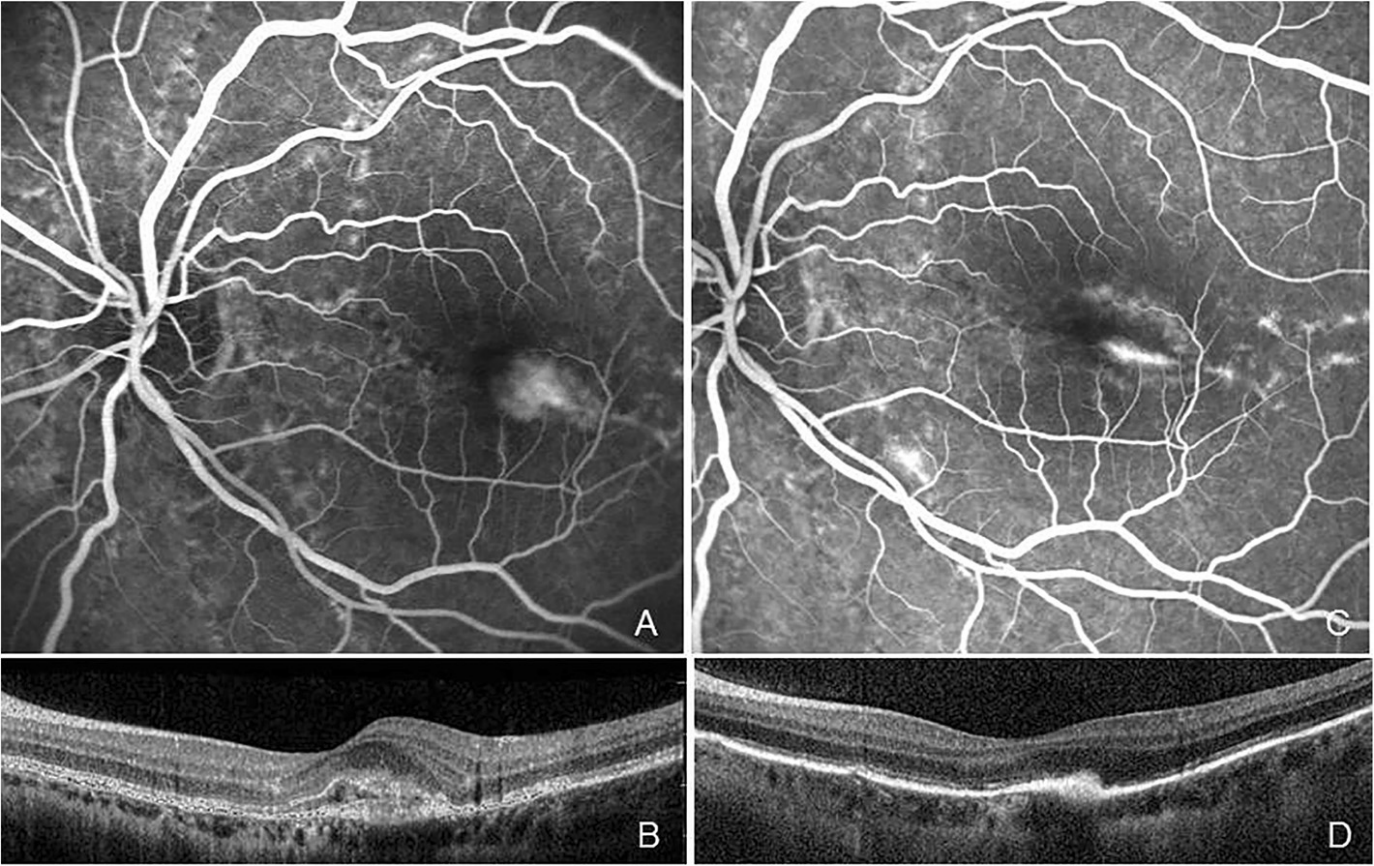
Fluorescein angiography (FA) late frame and spectral-domain optical coherence tomography (SD-OCT) scan showing choroidal neovascularization (CNV) in the paramacular area 2 months after the diagnosis of multiple evanescent white dot syndrome, corresponding to a zone previously occupied by outer retinal inflammatory signs (**a** and **b**). FA late frame and SD-OCT scan 1 month after intravitreal injection of ranibizumab, showing regression of the CNV (**c** and **d**)

Two months later, the patient returned because of sudden vision loss in her LE (20/100). FA indicated CNV in the paramacular area in LE. FA and ICGA showed no signs of choriocapillaris inflammation in BE (Fig. 3a, b). We proposed to the patient an intravitreal injection of ranibizumab as an off-label treatment option. She signed informed consent and was given a single injection of ranibizumab (0.5 mg/0.05 mL) following the normal procedure. One month after the injection BCVA improved up to 20/40, the CNV showed regression (Fig. 3c, d), and there was no need for retreatment up to her latest follow-up visit 1 year after the injection, when BCVA had improved up to 20/25.

ASs are often complicated by the appearance of CNV [2, 3]. To date there are no reports of AS associated with acute ocular inflammation. MEWDS is a unilateral inflammatory disease, which usually resolves spontaneously, with full recovery [11]. Here we describe a patient with ASs, who was diagnosed with coincident MEWDS. Two months after this diagnosis she developed CNV, which was effectively treated by intravitreal ranibizumab. In the current literature there are only six cases of CNV that developed after (from 4 weeks to 13 years) the diagnosis of MEWDS [6–10, 12]. Only two were effectively treated by intravitreal anti-vascular endothelial growth factor (VEGF) [10, 12]. In all other cases the visual loss was permanent despite treatment [6–9].

The case described is unusual: the CNV occurred in a patient with ASs, 2 months after the diagnosis of MEWDS. Although both ASs and MEWDS may contribute to the onset of CNV, the patient’s young age, good prognosis after intravitreal ranibizumab and the presence of MEWDS involving the macular area may indicate an inflammatory etiology of the CNV.

Though the pathophysiologic mechanism remains unclear, it has been suggested that alterations of Bruch’s membrane or the outer retinal barrier caused by choroidal inflammation may be associated with ischemic and/or inflammatory CNV [13]. It has also been hypothesized that the inflammatory processes induce the release of chemokines that favor angiogenesis [14]. This patient was in fact younger than the mean age at which CNV usually develops associated with ASs [15, 16]. Moreover, in this case a single intravitreal ranibizumab injection had a lasting effect (up to at least 1 year), as shown by FA and OCT, and by the maintenance of good BCVA.

Our results are in agreement with Rouvas et al. who have described the good responses to intravitreal treatment with ranibizumab for inflammatory retinal diseases [17]. This might be explained by the different nature of the CNV, in which inflammation may have played an important pathogenic role, compared to other CNVs secondary to ASs which tend to be associated with a worse visual prognosis, and need more injections [15].

## Conclusion

In this case of ASs, MEWDS completely resolved after 2 weeks, but 2 months later CNV developed. A single intravitreal injection of ranibizumab had a lasting effect. This illustrates the importance of correct etiological assessment of CNVs, for proper therapeutic management. Although both ASs and MEWS may have contributed to the CNV in our patient, an inflammatory pathogenesis must be considered, especially in young patients, where an idiopathic CNV may be the first manifestation of inflammatory chorioretinal diseases [18].

## Abbreviations

ASs, angioid streaks; CNV, choroidal neovascularization; MEWDS, multiple evanescent white dot syndrome; BCVA, best-corrected visual acuity; RE, right eye; LE, left eye; BE, both eyes; FA, fluorescein angiography; ICGA, indocyanine green angiography; SD-OCT, spectral-domain optical coherence tomography
